# Prenatal Ozone Exposure Induces Memory Deficiencies in Newborns Rats

**DOI:** 10.3389/fnmol.2019.00244

**Published:** 2019-10-15

**Authors:** Verónica Custodio, Carmen Rubio, Carlos Paz

**Affiliations:** Departamento de Neurofisiología, Instituto Nacional de Neurología y Neurocirugía, Mexico City, Mexico

**Keywords:** prenatally, ozone, memory, learning, NR2B, MAPK

## Abstract

Air pollution is fully acknowledged to represent a major public health issue. Toxic environmental substances, such as ozone, interfere with prenatal development. Animals exposed to ozone (O_3_) *in utero* develop biochemical and morphological alterations. This gas has been proven to decrease cognitive capacity in different species. In the present study, we assessed the possible alterations in memory and spatial learning in the offspring of female rats who were exposed to 1.0 ppm of O_3_ embryonic development. Two instruments were used to evaluate possible alterations: the T-maze and a Skinner box. MAPK, ERK, p-ERK, and NR2B proteins, which are widely regarded as responsible for the learning process in the hippocampus and cortex, were also assessed by immunohistochemistry. We found that male rats exposed to O_3_ *in utero* displayed a significant delay to reach the correct response using the spatial learning test as compared to the control group. The female rats exposed to O_3_ showed a significant delay to reach the correct response as compared to the female control group in the Skinner box. We also found that while the male rats showed decrease in significant differences in the expression of NR2B, ERK and increase in MAPK. Females only showed increase in MAPK, p-ERK and decrease in ERK, when compared to their respective control group. It is possible that the deficits are associated to hormonal expression, inflammation and oxidative stress alterations. In summary, these results suggest that exposure to O_3_ can interfere with prenatal development, resulting in learning and memory deficiencies in rats.

## Introduction

Air pollution is a major public health issue. The global burden of disease attributed to environmental factors is estimated to be 25%, affecting 35% of children under 5 years of age (Prüss-Ustün et al., 2016). Ozone (O_3_) is one of the most common environmental pollutants. The exposure to O_3_ has been associated to damage and loss of spatial memory in rodents. The dorsal hippocampus participates in learning processes and spatial memory, specifically regions CA1, CA3 and the dentate gyrus (Gilbert and Brushfield, 2009). These regions have been found to differ in male and female rats during postnatal development, the difference has been attributed to the role of sex hormones (Roof, 1993; Woolley and McEwen, 1993). Adult rats exposed to 1.0 ppm of O_3_ for 4 h show a significant decrease in the number of dendritic spines of pyramidal neurons in the CA1 region of the hippocampus, which correlates with memory deterioration (Avila-Costa et al., 1999). Nevertheless, an increase in the length of dendritic spines, as well as conformational changes after the induction of long-term potentiation (LTP), can provide a structural basis for memory (Bliss and Collingridge, 1993; Hosokawa et al., 1995). On the other hand, it is known that the synaptic plasticity and LTP implicated in learning and memory occur mainly through glutamatergic receptors *N*-methyl-D-aspartate (NMDA) located in the hippocampus and cerebral cortex (Muller and Lynch, 1988). This heterotetrameric protein complex consists of two required NR1 subunits and two of four possible NR2 subunits: NR2A, NR2B, NR2C, and NR2D. The NR2 subunits in the hippocampus and adult cortex are generally NR2A and NR2B. The NR2B subunits are present in the brains of mutant mice and are known to be the speed limiting factor in the control of NMDAR-mediated synaptic plasticity and memory formation called LTP (Li and Tsien, 2009). MAPKs are a group of protein kinases that participate in LTP. Prior research has determined that MAPKs, ERK1, and ERK 2, located in the hippocampus of rodents, play a significant role in neuronal plasticity and thus, memory processes (Besnard et al., 2013). The administration of (PD 098059), an inhibitor of ERK, results in amnestic processes that indicate that the activation of ERK is necessary for the consolidation of memory (Duvarci et al., 2005; Miller and Marshall, 2005; Cestari et al., 2006; Krawczyk et al., 2016). Several studies have reported that children exposed to air pollutants, including fine particles and O_3_, develop systemic inflammation, attention deficit and memory deterioration (Calderón-Garcidueñas et al., 2008, 2016). Memory processes depend on the sleep-wake cycle (Fraize et al., 2016). It is widely known that sleep disorders affect both memory and learning. Previously, we demonstrated that O_3_ exposure induces a significant reduction in rapid eyes movements (REM) sleep in both adult rats and pups prenatally exposed to this gas (Haro and Paz, 1993; Paz, 1997). Therefore, we assume that animals exposed to O_3_ during gestation will present disruptions in proteins related to learning and memory processes. In order to verify this, we performed immunohistochemical localization of NR2B, MAPK, and ERK, that participate in the memory process that were measured by immunohistochemistry. We used the T maze and Skinner’s box to investigate cognitive and memory changes. The behavior tests are an instrument designed to analyze processes such as learning and memory, stimulus discrimination, of which are involved in the maturation of cellular components and circuits associated to the hippocampus and cortex.

## Materials and Methods

All procedures followed the International Guide for the Care and Use of Laboratory Animals (NIH publication No. 86-23, revised 1987). This study was carried out in accordance with the principles of the Basel Declaration and recommendations of the Institutional Review Committee according to the current Mexican legislation norm (NOM-062-ZOO-1999), Institutional Committee for the Care and Use of Laboratory Animals. The protocol was approved by the Institutional Research Committee. All efforts were made to minimize both the number of animals used and any potential pain or distress.

### Animals and Nursing Procedures

As we have described previously (Custodio et al., 2010). In summary, six female Wistar rats with an average weight of 300 ± 20 g were housed overnight with males, the presence of sperm in a vaginal smear the following day indicated the first day of pregnancy. The animals were weighed throughout the gestation period, we registered the corporal gain of both groups, without observing significant differences, confirming previous results. The rats were then transferred to hermetic chambers supplied with unpolluted air (1.7 l/min) and maintained at a constant temperature (23^°^C), humidity (70%), with free access to food and water under a 12/12 light-darkness cycle (control group *n* = 3). Additionally, pregnant rats were housed in the above conditions, but the air was polluted with 1.0 ppm of O_3_ during the darkness phase throughout the first 20 days of gestation using an O_3_ generator (Triozon P15). The concentration of O_3_ was measured and maintained at a constant rate using a Serinus 10 ultraviolet light analyzer (O_3_ group *n* = 3). We used higher doses than those associated with human toxicity because, according to previous reports, rats usually display higher concentrations of endogen antioxidants (Slade et al., 1993; Kari et al., 1997). When the offspring of both groups were born, they were kept in pollutant-free air conditions and stayed with their mothers until weaning (postnatal day 21). Female and male rats were used for two behavioral tests, T-maze (postnatal day 30) and Skinner’s box (postnatal day 60). The control group included 16 of their offspring, eight males and eight females. For the ozone group we included 18 offspring, 10 males and 8 females. Once the behavioral evaluations were concluded, the rats were sacrificed in order to perform immunohistochemical quantification of NR2B, MAPK, ERK, and p-ERK.

### T-Maze Spatial Learning

The tests associated with tasks of spatial alternation or conditioned choice have been used as tools for the study of behavior and animal cognition. Once the offspring were 30 days old, they were subjected to partial caloric deprivation (85%) of their ideal caloric intake. The purpose was to stimulate appetite and therefore the search for food through the T-maze, which consists of a starting area, an exit gate, a straight central corridor (72 cm) and a bifurcation with two left and right arms, the walls of the labyrinth are 30 cm high. During the first week, the rats were placed in the starting area, immediately afterward, the gate was opened so that they could explore the T-maze. Foam was placed on the floor of the corridor so that the rats could associate the soft texture with the presence of food in the right arm of the T-maze. Every day the tests were carried out between 9:00 and 13:00 h. The number of hits and the time they took to reach the right arm were quantified. In order to be considered correct, the rat had to reach the food within 1.2 min of the starting point. If it exceeded this time, it was considered incorrect. Once the rats managed to perform 75–100% of correct responses for five consecutive days, the foam placed on the floor of the corridor was replaced by sandpaper (rough texture). When the rats managed to perform 75–100% of hits for five consecutive days, the rats associated the presence of food with the left arm. The final test consisted of alternating the two stimuli (foamy and sandpaper). We alternated between foam and sandpaper an equal number of times, avoiding to establish a pattern. Six sessions were held and each session was made up of eight events, one session per day. A different foam was used for females and males as we sought to avoid some bias in the performance of the rat due to hormonal factors. Between subjects, the foam and the T-maze were thoroughly cleaned with a solution of water, alcohol and dextran so that the smell of the previous rat would not distract the next rat in line to perform the test.

### Skinner’s Box (Acquisition of Temporary Control in the Fixed Interval Program)

Habituation: On the 60 postnatal day, rats were placed in the operant conditioning chamber (305 mm wide × 350 mm long × 365 mm high) for 30 min. The chamber was equipped with a response lever and a food dispenser. The rats were conditioned so that when they pressed the lever, they obtained food (0.08 g) that worked as a reinforcer to consolidate the behavior and increase the likelihood that it would be recurrent. The next 5 days was reinforced if rat pressed the lever. At the beginning of the test, the rats were subjected to partial caloric deprivation (85%). 30 reinforcers were offered per session, which were provided as long as the rats discriminated that when pressing the lever, they had to pass fixed intervals of 10 s, which were quantified to obtain the percentage of correct responses during the six sessions.

### Immunohistochemistry

To determine the expression of the NR2B subunit of the receptor to NMDA, MAPK, ERK, and p-ERK, in layers 3–5 characterized by presenting external and internal pyramidal cells and granular cells of prefrontal cortex and pyramidal cells of CA1 and CA3 of hippocampus from each group were anesthetized and sacrificed after to behavioral test. As we have described previously (Martínez-Lazcano et al., 2018). Briefly, once anesthetized with sodium pentobarbital (63 mg/kg), the rat was transcardially injected with heparin (5000 U/ml) and then perfused through the right ventricle with phosphate-buffered saline (PBS) for 30 s, followed by 4% paraformaldehyde dissolved in PBS (pH = 7.2) at 4°C. Brains were removed from skulls, fixed for 48 h in buffered paraformaldehyde and dehydrated with alcohol and xylene. Then, the brains were embedded in paraffin and sectioned parasagittally (7 μm) using a microtome. Sections were baked overnight at 65°C followed by deparaffinization with xylene and rehydration through a graded series of ethanol to PBS. Slides were treated with sodium citrate (10 mmol/L in PBS, pH 6.0) for 20 min at 95°C, and endogenous peroxidase was blocked with 3% hydrogen peroxide for 10 min at room temperature. Sections were then rinsed in PBS and incubated for 24 h at 4°C with NR2B (BML-SA426-0050 ENZO), p-ERK (sc-7383 Santa Cruz Biotechnology, United States), ERK (sc-1647 Santa Cruz Biotechnology, United States) or MAPK (sc-271684 Santa Cruz Biotechnology, United States) antibodies at a final dilution of 1:100. After three rinses, the sections were incubated with a secondary anti-goat IgG conjugated with rhodamine (1:100 Jackson) and IgG conjugated with fluorescein (1:100 Jackson), respectively for 2 h at 4°C. Finally, the sections were mounted with DAPI-stained nuclei (Fluroshield F6057 in blue, Sigma, MO, United States). Slides were observed using an Olympus IX81-F3 microscope (Olympus Corporation, Tokyo, Japan) equipped with an Olympus evolution Q-imaging digital camera kit. Immunopositive cells were quantified in three different areas. Therefore, we tried to quantify the same area in all three sections of Ammon’s horn. In section CA1, the proximal zone corresponds to field 1, the medial zone is field 2 and the posterior zone is field 3. Correspondingly, in section CA3, the proximal zone is field 1, the medial zone is field 2 and the posterior zone is field 3. We also quantified 3 fields of the prefrontal cortex, the most anterior is field 1, the medial zone is field 2 and the subsequent is field 3. These sections were photographed and analyzed with Image-Pro Plus software Version 7.0. The images were obtained with a 40× objective in three representative 520-mm^2^ fields, under the same light and processing conditions. The images were filtered with Image J^®^ (NIH, Bethesda, MD, United States) software to remove the background using a minimum filter (2.0 pixels), and the mean of the integrated optical density (mean IOD), we have previously reported as IOD arbitrary units and calculated based on the staining intensity for each pixel (Ballesteros-Zebadua et al., 2014). To illustrate the results, photographs were taken with a 60× lens plus a 1.6× zoom.

### Statistical Analysis

Statistical analyses were conducted using SPSS software (version 20). A repeated measures ANOVA model was used to analyze T-maze to correct response duration, Skinner’s box, two-factor sex (males and females) and treatment (control and experimental) data. T-maze to latency of correct response and optical density of immunohistochemistry was conducted as one-way ANOVA. Tukey post-test considering ^∗^*P* < 0.05. All data were expressed as the mean ± SE.

## Results

### T Maze

**FIGURE 1:**
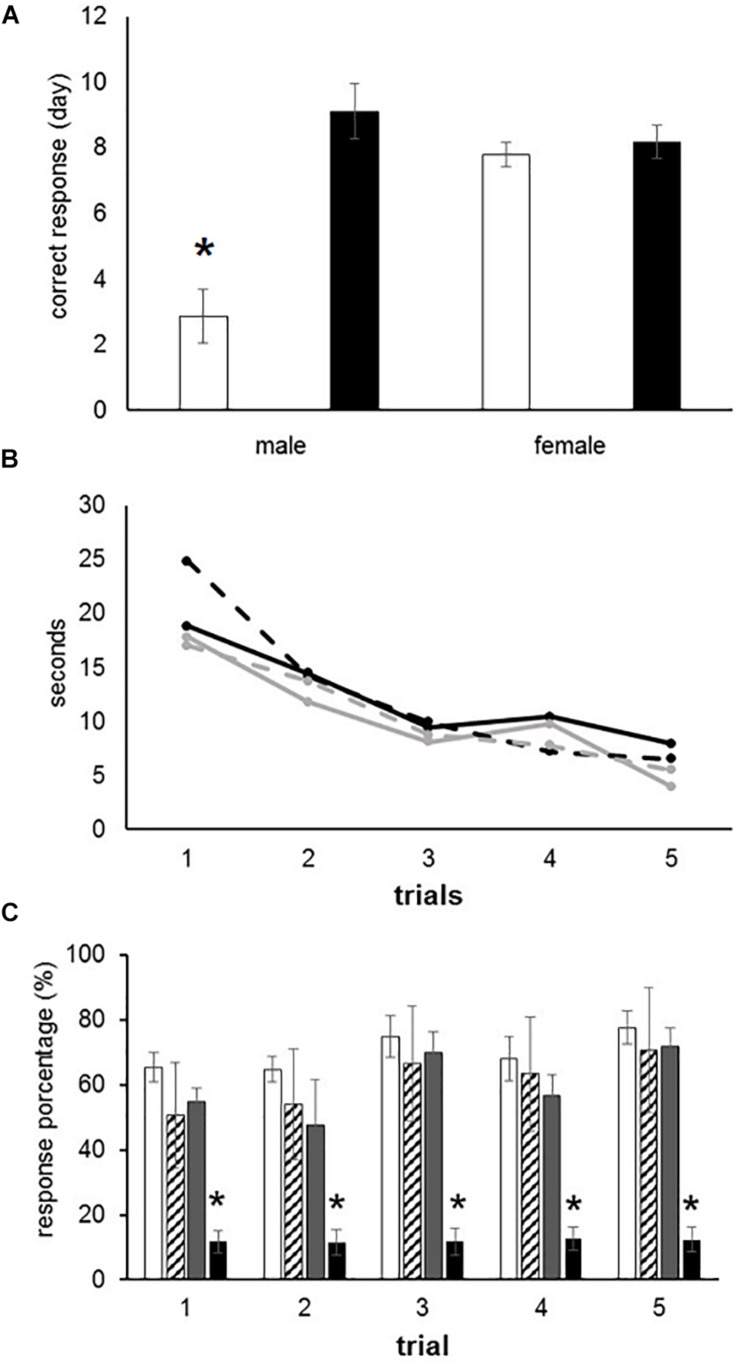
Learning deficits in prenatally ozone exposed rats. **(A)** T-maze test showing a significant increased latency to reach the correct response in ozone exposed males as compared to control (black bars) using one-way ANOVA comparison statistics. **(B)** Progressive improvement to find the recompense in the T-maze test without differences between groups. **(C)** Significant reduction to reach the correct response in female rats exposed to ozone using the Skinner box. The bars indicate the means ± SEM, using the repeated measures ANOVA. ^∗^*P* ≤ 0.05.

The male offspring exposed to O_3_ during gestation exhibited an increased response latency during 75–100% of the hits per trial, when compared with control males (*F*_(__3__,__28__)_ = 16.16, *P* < 0.000). Conversely, female offspring exposed to O_3_ *in utero* did not present significant differences when compared to females in the control group. Male offspring who were part of the control group performed spatial recognition tests with a higher efficiency rate than female offspring in the same group, thus supporting previous studies that describe cerebral sexual differentiation (Figure 1A). After analyzing the average time each group (females exposed to O_3_, female control group, males exposed to O_3_ and male control group) took to obtain 75–100% correct responses, we found no statistically significant differences when compared per day. However, we observed a gradual decrease in the learning curve, which is expressed in seconds, in all four groups (*F*_4_ = 3.399) (Figure 1B). When the rough texture (sandpaper) was placed in the long corridor and the reinforcement (pellet) in the left arm, the latency to obtain 75–100% of correct responses was immediate. However, no statistically significant differences were found in females and males exposed to O_3_ compared to those in the control group. In the alternation analysis, we did not find significant differences when we compared males with females (data not shown).

### Operant Conditioning (Skinner Box)

Rats were placed in an operant conditioning chamber and received positive reinforcement in order to distinguish a fixed interval of 10 s. The subjects had to press a lever during 30 events in 10-s intervals in order to receive a reward (pellet). This behavior was tested during five consecutive days. We found no statistically significant differences between males exposed to O_3_ and the male control group. However, females of the experimental group performed worse than the female control group (*F*_3_ = 8.212, *P* < 0.001), as shown by the analysis of variance test (Figure 1C).

### Expression of NR2B, MAPK, ERK, and p-ERK

**FIGURE 2:**
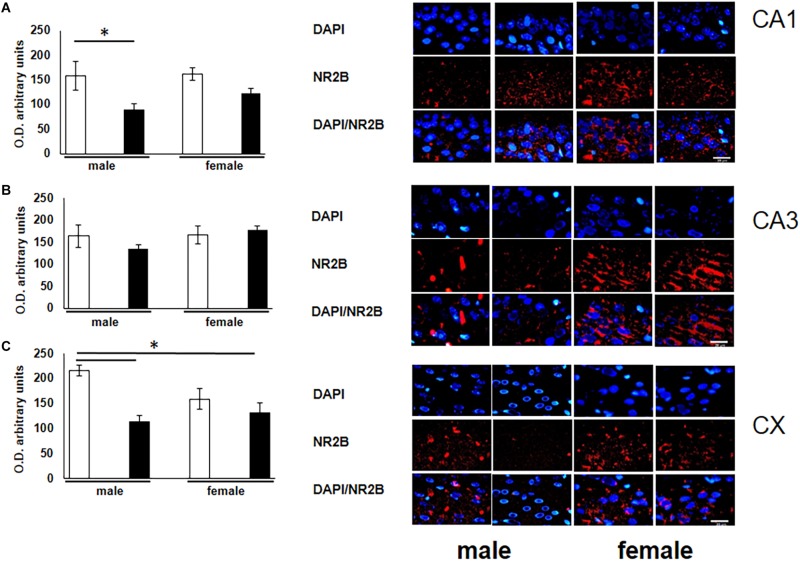
Representative microphotography (objective 60× and zoom 1.6×) showing immunopositivity for the NR2B subunit of the NMDA receptor (red) and DAPI-stained nuclei (blue). The graph indicates optical density arbitrary units for control group (white bars) and ozone group (black bars) in males and females, **(A)** CA1, **(B)** CA3 and **(C)** CX. We observed a decrease in the expression of NR2B in three structures (CA1, CA3, and CX), when compared with their respective controls. Bars indicate the mean ± SEM, using one-way ANOVA comparison statistics. ^∗^*P* ≤ 0.05. Scale bar = 20 μm.

To corroborate the possible alterations of the glutamate receptor subunit in rats exposed to O_3_ during gestation, we quantified the expression of this receptor in structures that participate in learning and memory. A significant decrease of NR2B expression was observed in the CA1 subfield of the hippocampus in male subjects exposed to O_3_ when compared with their respective controls (Figure 2A). In addition, we observed a decrease of NR2B expression in the prefrontal cortex of both males and females exposed to O_3_ (Figure 2C).

**FIGURE 3:**
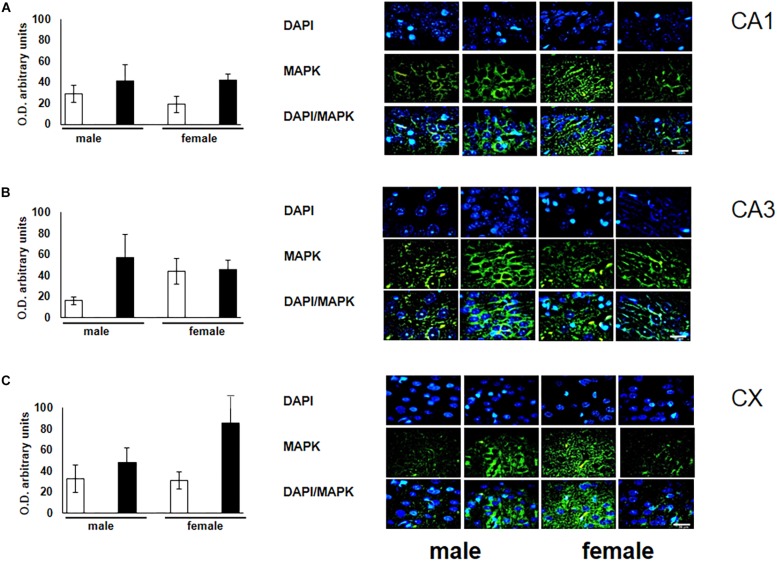
Representative microphotography (objective 60× and zoom 1.6×) showing immunopositivity for the MAPK (green) and DAPI-stained nuclei (blue). The graph indicates optical density arbitrary units for control group (white bars) and ozone group (black bars) in male and females, **(A)** CA1, **(B)** CA3 and **(C)** CX. We observed a non-significant increase in the expression of MAPK in CA1, CA3, and CX. Bars indicate the mean ± SEM, using univariate comparison statistics. ^∗^*P* ≤ 0.05. Scale bar = 20 μm.

**FIGURE 4:**
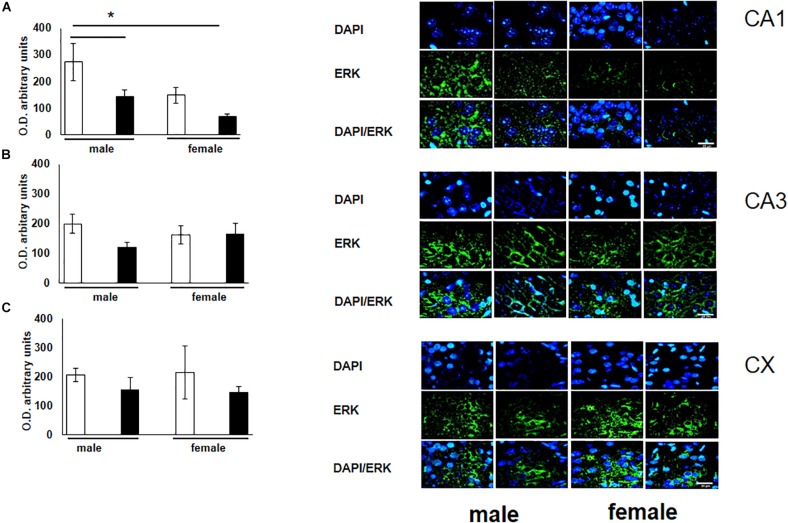
Representative microphotography (objective 60× and zoom 1.6×) showing immunopositivity for the ERK (green) and DAPI-stained nuclei (blue). The graph indicates optical density arbitrary units for control group (white bars) and ozone group (black bars) in male and females, **(A)** CA1, **(B)** CA3 and **(C)** CX. We observed a statistically significant decrease in the expression of ERK in the CA1 subfield in both males and females exposed to ozone. Bars indicate the mean ± SEM, using univariate comparison statistics. ^∗^*P* ≤ 0.05. Scale bar = 20 μm.

**FIGURE 5:**
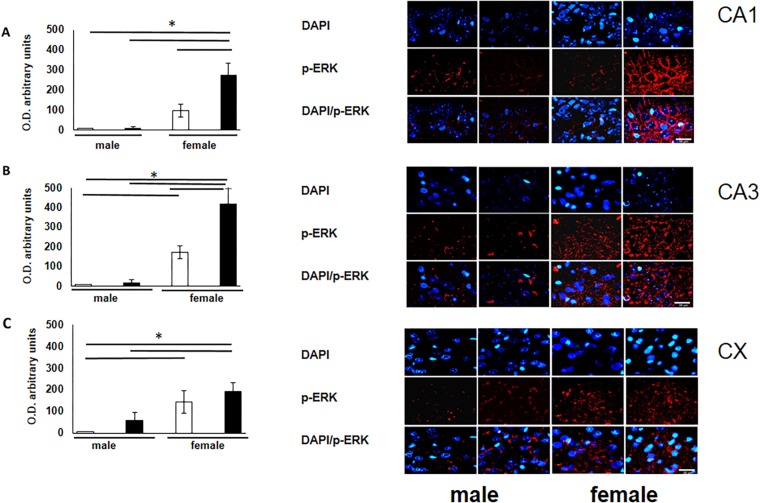
Representative microphotography (objective 60× and zoom 1.6×) showing immunopositivity for the p-ERK (red) and DAPI-stained nuclei (blue). The graph indicates optical density arbitrary units for control group (white bars) and ozone group (black bars) in male and females, **(A)** CA1, **(B)** CA3 and **(C)** CX. Expression of p-ERK is significantly increased in three structures (CA1, CA3, and CX). Bars indicate the mean ± SEM, using univariate comparison statistics. ^∗^*P* ≤ 0.05. Scale bar = 20 μm.

To verify the participation of the MAPK family in learning and memory processes, we evaluated the expression of MAPK, ERK, and p-ERK in the hippocampus and prefrontal cortex. We observed an increase (not statistically significant) in the expression of MAPK in subfields CA1 and CA3, as well as in the prefontal cortex of male and female experimental groups when compared with their respective controls (Figures 3A–C). A statistically significant decrease in the expression of ERK was observed in the CA1 subfield in both males and females exposed to O_3_ (Figure 4A). We did not observe significant differences in the CA3 subfield and cortex (Figures 4B,C). Our results show that the expression of p-ERK is significantly increased in the CA1 and CA3 subfields of the hippocampus in experimental female rats when compared with the control group of both sexes and with experimental males exposed to ozone. In experimental males, a significant decrease was detected in the CA3 subfield and prefrontal cortex when compared to experimental females (Figures 5A–C).

## Discussion

Ozone exposure produce central nervous system biochemical, morphological and physiological disturbance changes both in humans and experimental animals. In the present study, we observed that prenatal exposure to O_3_ in male and female rats lead to a deficit in differential cognitive abilities. Learning and memory processes are an example of nervous system plasticity associated to molecular change. Our findings demonstrate that male rats exhibit a significant decrease in the expression of NR2B and ERK, as well as an increase in MAPK. Likewise, females exhibit an increase in MAPK and p-ERK, and a decrease in ERK when compared to their control group. Previous studies have proven that NR2B subunits, which are abundant in the adolescent brain, enhance synaptic plasticity (Li and Tsien, 2009; Wang et al., 2014).

Prior research has been made to evaluate memory a one trial passive avoidance conditioning and lipid peroxidación in male adult rats exposed to different concentrations of O_3_ by 4 h, found loss of memory retention and lipid peroxidation most in hippocampus and cortex (Dorado-Martínez et al., 2001). Breathing clean air during pregnancy is important for fetal growth. Air pollution can affect maternal respiratory function or general health, which in turn can affect uteroplacental and umbilical blood flow, all of which are important determinants of fetal growth (Vorherr, 1982). Air pollution causes decrease in the caliber of placental blood vessels (Veras et al., 2008). During the present study, rats were exposed reactive oxygen species and other inflammation products *in utero* for 21 days, which resulted in structural damage to the hippocampus and cortex. We previously found that rats exposed to O_3_ during pregnancy showed noteworthy inconsistence in the cerebellar foliation and necrotic evidences on the surviving cells (Rivas-Manzano and Paz, 1999). Preservation of the dendritic tree is imperative since it is a site of neuronal junctions and receptors responsible for plastic processes such as LTP. Our analysis of the immunohistochemical quantification evidence a decrease of the subunit of the NR2B in the individuals exposed to O_3_ (Figures 2A–C). This data correlates with the learning and memory alterations described in this manuscript. Prenatal exposure to O_3_ possibly modified the processes of migration, maturation and neurogenesis that occur during embryonic development. One of the mechanisms by which they do not develop adequately is the lack of neurotransmitters that participate as trophic factors. We have investigate previously reported that the decreased concentration of noradrenaline in the cortex and cerebellum caused chronic damage. With our findings we reiterate that the hippocampus is one of the structures most susceptible to damage as shown in our behavior and immunohistochemical results, which exhibits a decrease in the expression of the MAPK and the NR2B receptor subunit signaling pathways, whose participation has been widely described in the plastic processes of learning and memory (Ribeiro et al., 2005). Moreover, is has been previously reported that lesions in the hippocampus cause cognitive deterioration (Rahmati et al., 2019). Blood vessels provide the brain with oxygen and nutrients necessary for the development and maintenance of cerebral function, cerebral angiogenesis is intimately associated with these processes (Wittko-Schneider et al., 2014). Astrocytes, microglia, neurons, endothelial cells and pericytes interact together and are called neurovascular units (NVU). NVUs are responsible for keeping the brain free of toxins or pathogens (Andreone et al., 2015). Calderón-Garcidueñas et al. (2016) reported that children and dogs living in Mexico City who were chronically exposed to polluted air, presented alterations of NVUs. Among these alterations were the accumulation of perivascular lipids, abnormal basal cerebrovascular membranes and the presence of ultrafine particles in mitochondria, basal membranes, axons and dendrites of white matter in the prefrontal cortex. The prefrontal cortex is related to memory, its alteration is associated with cognitive and behavioral disorders such as schizophrenia and attention deficit disorder (Goldman-Rakic, 1996; Sullivan and Brake, 2003). In the early stages of mammal development, plasticity processes occur in the nervous system (Lledo et al., 2006; Kim et al., 2012), which can be altered by prenatal exposure to O_3_ (Rivas-Manzano and Paz, 1999; Custodio et al., 2010). Adult Wistar rats, exposed to 1.0 ppm O_3_ for 4 h, show a significant decrease in the number of dendritic spines of pyramidal neurons in the CA1 area of the hippocampus, data that correlates with memory deterioration (Avila-Costa et al., 1999). The increase in the length of dendritic spines, as well as conformational changes after the induction of LTP, can provide a structural basis for memory (Bliss and Collingridge, 1993; Hosokawa et al., 1995). Ozone exposure causes a deterioration in the plastic mechanisms that participate in the plasticity necessary for memory processes, since these data correlate with the results of the present manuscript, decrease in the subunit of the NR2B and ERK receptor in the hippocampus and cortex of males and females exposed prenatally to ozone.

Oxidative stress has been recognized as a mechanism through which damage may develop after O_3_ exposure (Paz, 1997; Rivas-Arancibia et al., 2010; Martínez-Lazcano et al., 2013, 2018; González-Guevara et al., 2014). When comparing the immunological changes in the bronchoalveolar lavage fluid of rats and humans it is observed that 2.0 and 0.4 ppm of O_3__,_ respectively, are required to obtain similar effects (Hatch et al., 1994). Several studies agree that chronic oxidative stress is likely to trigger neurodegenerative diseases such as amyotrophic lateral sclerosis, Alzheimer’s, Parkinson’s and other disorders associated with memory loss (Dorado-Martínez et al., 2001). Chronic exposure to low doses of O_3_ (0.25 ppm) for 4 h a day induces a state of oxidative stress and irreversible progressive neurodegeneration (Rivas-Arancibia et al., 2010). In the hippocampus, this process is characterized by mitochondrial alterations that result in energy deficit, endoplasmic reticulum stress and alterations of the Golgi apparatus, nuclear changes, activation and proliferation of the glia, cell death by apoptosis and necrosis, the intracellular accumulations of beta-amyloid, as well as the deficit in the reparative response of the brain and the alterations in memory and learning processes (Rivas-Arancibia et al., 2016). Neurotransmitters have a trophic function during the nervous system development. Noradrenaline participates in proliferation, cell maturity and neural architecture configuration (Tetsuro et al., 1977; Lauder and Bloom, 1979; Saito et al., 1996). The noradrenergic neurons of the locus coeruleus appear between day 10 and 13 of gestation, while the production of noradrenaline is first detected on pre-natal day 14. Our research group has demonstrated that exposure to 1.0 ppm of O_3_ during pregnancy induces an irreversible decrease in norepinephrine concentration in the cerebellum and cortex (Custodio et al., 2010).

Deficits in learning test parameters after O_3_ exposure could be attributed to morphological, chemical and anatomical alterations. The existence of sexual differentiation in learning processes has been evidenced after spatial orientation tests conducted in both male and female rats (Jost, 1983; Roof, 1993; Galea et al., 1994). These findings have been demonstrated using Morris’s water maze in 21-day-old rats (Roof, 1993). Even more the gonadal steroids, probably the testosterone metabolite estradiol, cause organizational effects during perinatal development which have multiple effects on the associational-perceptual-motor biases that guide visuospatial navigation (Williams et al., 1990; Williams and Meck, 1991). Sex-related differences in learning processes were observed through the activity of cytochrome oxidase, considered an indirect marker of neuronal activity. The conducted T-maze tests and observed that, in males, structures associated to spatial trajectories (infralimbic cortex and preliminary cortex) were inactive. Conversely, in females, structures related to spatial processing in the hippocampal system (supramammillary nucleus, nucleus accumbens, and ventral tegmental area) were active. The hippocampus is the cerebral structure most closely related to learning processes, its development extends to maturation. Mitosis in the granular cells of the dentate gyrus in rats takes place postnatally and continues in the adult period (Bayer, 1982). Previous studies have proven this brain region is different during post-natal development in male and female rats. This differentiation has been attributed to the role of sex hormones (Roof, 1993; Woolley and McEwen, 1993).

On the other hand, Jenkins and Dallenbach (1924) observed that university students enhanced their ability to recall different sets of words if, in the interval between learning and review, they were allowed to sleep. Retention decreased if the person was awake and active during the interval. McGeogh and Irion (1932), was among the first to quantify the beneficial effect of sleep on memory with classic verbal list learning experiments and estimated that the savings after sleep was 33%. Both experimental and clinical studies have shown that sleep has positive effects on different types of memory. Our group reported that O_3_ exposure lead to a significant decrease in REM sleep, this was observed in rats (Paz and Huitron-Resendiz, 1996; Rubio and Paz, 2003) and cats (Paz and Bazan-Perkins, 1992) exposed to concentrations of 0.2 to 1.2 ppm of O_3_. A permanent decrease in REM sleep was found in rats 30, 60, and 90 days of age studied in an environment free of pollutants but whose mothers were exposed 12 h a day to 1.0 ppm of O_3_ throughout pregnancy (Haro and Paz, 1993). Non-rapid eye movement sleep is increased by the enzyme cyclooxygenase which is regulated by the proinflammatory cytokines IL-1 and TNF (Krueger, 2008; Clinton et al., 2011).

Our findings show male rats perform better on spatial learning tests (T-maze test) when compared to control females. Under experimental ozone exposure conditions, we found that males increased latency to obtain 75–100% accuracy, which correlates with the decrease in the expression of the NR2B receptor. The females exposed to O_3_ did not present significant differences in latency, nor in the expression of this receptor, probably because estrogens protect hippocampal and cortex integrity (Figures 1A, 2A). Our results can be attributed to the lower expression of ERK in the hippocampal areas and cortex. On the other hand, the expression of p-ERK showed a significant increase in females with O_3_ exposure, which could probably be related difference in performance of these in the Skinner box. It is known that females present a higher expression of p-ERK under control conditions by estrogen regulation (Jiu-Qing et al., 2017).

The panorama obtained from these studies is not encouraging since exposure to O_3_ is a reality that is experienced day to day in metropolitan areas. Recent evidence suggests that MAP kinases ERK1, ERK2, and p38 mediate LTP processes, which are involved in the transcription of O_3_ induced IL-8, thus hypothesizing that differences in their activation they can control the interindividual variability of IL-8. It has been observed a significant correlation between the phosphorylation of ERK1/2 and the expression of IL-8, suggesting that ERK1/2 modulates the ozone-response mediated by IL-8 (Bowers et al., 2018). Ozone is a highly reactive gas unable to reach the developing tissues. However, in our laboratory we testify that the inflammation that this gas causes in the respiratory system is capable of affecting distant organs (Martínez-Lazcano et al., 2018). Thus, we conclude that prenatal O_3_ exposure interfere with the brain development and consequently with memory, learning and metabolic pathway deficiencies of MAPKs in rats. However, additional studies are required to address how prenatal exposure to O_3_ and thus, the interaction between inflammatory processes, oxidative stress, hormones and abnormal brain development affect learning and memory.

## Data Availability Statement

The datasets generated for this study are available on request to the corresponding author.

## Author Contributions

VC and CR were responsible for performing all the experimental procedures, as well as obtaining all the resulting data and contributing to its analysis, and contributed to the plan and design of the experiments, as well as to the writing and revising of the manuscript. CP performed the original plan and design of the experiments, contributed to the analysis of all the resulting data, as well as to the writing and revising of the manuscript.

## Conflict of Interest

The authors declare that the research was conducted in the absence of any commercial or financial relationships that could be construed as a potential conflict of interest.
